# Nuestra experiencia en el tratamiento de las malformaciones linfáticas: Lecciones y protocolización

**DOI:** 10.23938/ASSN.1127

**Published:** 2025-09-17

**Authors:** Esther Comajuncosas Pérez, Julio César Moreno-Alfonso, Ada Yessenia Molina Caballero, Alberto Pérez Martínez

**Affiliations:** Servicio Navarro de Salud-Osasunbidea Hospital Universitario de Navarra Servicio de Cirugía Pediátrica Pamplona Navarra España

## Sra. Editora:

Las malformaciones linfáticas son anomalías vasculares caracterizadas por conductos linfáticos displásicos y cavidades quísticas llenas de linfa, cuya causa exacta es desconocida en la mayoría de los casos. Generalmente son asintomáticas y solo precisan observación; la necesidad de tratamiento viene determinada por su localización en zonas críticas (compromiso vital), por repercusión funcional o por causar una deformidad corporal evidente con impacto psicosocial^1^. La extirpación quirúrgica ha sido considerada la terapia de elección para estas malformaciones, aunque son intervenciones agresivas que no siempre consiguen la curación del paciente^1^. En los últimos años el tratamiento ha evolucionado sustancialmente gracias al desarrollo de la inmunoterapia y la radiología intervencionista, consiguiendo resultados comparables a la cirugía^2^.

Presentamos los resultados de una cohorte infanto-juvenil (0-15 años) con malformaciones linfáticas, tratada entre 2006-2022 en el Hospital Universitario de Navarra (Pamplona, España), centro de referencia autonómica para el tratamiento de las anomalías vasculares, con el objetivo de identificar puntos de mejora y establecer un protocolo de atención.

Inicialmente, el tratamiento fue o escleroterapia con OK-432, y actualmente desde 2022 y de forma protocolizada desde 2024, con bleomicina o doxiciclina, o bien cirugía si las dos primeras líneas de tratamiento no resultan efectivas. Se analizaron variables demográficas (sexo masculino/femenino, edad en años), clínicas (diámetro en cm, localización: cervical, pared abdominal, mesenterio, tórax, extremidad superior o inferior, axilar, retroperitoneal; tipo: mixta, macroquística, microquística, sin establecer; sintomatología sí/no) y los desenlaces existencia de complicaciones sí/no y curación sí/no.

Se incluyeron 23 niños con una edad media de 4,9 años (DE=3,5), y la mayoría de sexo masculino (65,2%). El diámetro medio de las malformaciones linfáticas fue de 6,6 cm (DE=4,2) y la localización más frecuente fue el cuello (47,8%) seguida por la pared abdominal (13%). Más de la mitad se encontraban asintomáticos (52,2%), y la principal manifestación clínica fue la infección (17,4%), seguida de sangrado (13%), dolor (8,7%), dolor y edema tras traumatismo (4,3%) y un cuadro de suboclusión intestinal (4,3%) (Tabla 1).

**Tabla 1:** Características de la muestra pediátrica estudiada (n=23) y de sus malformaciones linfáticas

Variable	n (%)
Sexo masculino	15 (65,2)
Edad (años), *media (DE)*	4,9 (3,5)
Diámetro (cm), *media (DE)*	6,6 (4,2)
*Localización*	
Cervical	11 (47,8)
Pared abdominal	3 (13,0)
Mesenterio	2 (8,7)
Tórax	2 (8,7)
Extremidad superior	2 (8,7)
Extremidad inferior	1 (4,3)
Axilar	1 (4,3)
Retroperitoneal	1 (4,3)
*Tipo de malformación linfática*	
Mixta	4 (17,4)
Macroquística	1 (4,3)
Microquística	3 (13)
Sin establecer	15 (65,2)
*Sintomatología*	
Asintomáticos	12 (52,2)

DE: desviación estándar.

**Figura 1:**
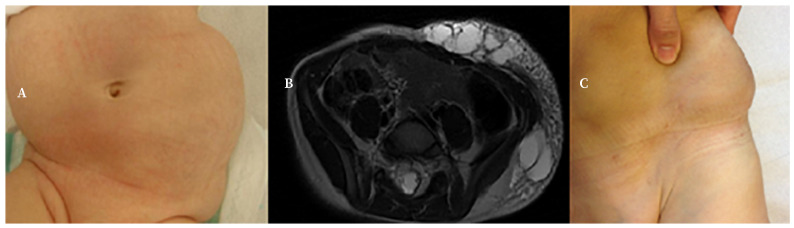
Lactante de 2 meses de vida con malformación linfática mixta de la pared abdominal. **A**. En el momento del diagnóstico. **B**. Resonancia magnética realizada a los 4 años de edad, previa a la escleroterapia, donde se aprecia extensa afectación del plano subcutáneo del hemiabdomen izquierdo, con múltiples imágenes tabicadas conformando cavidades, siendo las mayores a nivel del flanco de 2-3 cm de volumen. **C**. Fotografía clínica a los 5 años de vida después de tres sesiones de escleroterapia con bleomicina y extirpación quirúrgica parcial de la malformación.

Se realizaron 33 procedimientos terapéuticos (1,4 por paciente). El primer procedimiento consistió en extirpación quirúrgica (n=13; 56,5%), o escleroterapia con OK-432 (n=4; 17,4%), con bleomicina (n=4; 17,4%) o con doxiciclina (n=2; 8,7%). Cuatro casos (17,4%) precisaron un segundo procedimiento (tres esclerosis y uno cirugía); y solo un niño con una gran malformación en la pared abdominal con un espesor máximo al nacimiento de 5,2 cm, precisó ocho intervenciones (tres procedimientos de escleroterapia con OK-432, tres esclerosis con bleomicina y dos cirugías para extirpación) (Fig. 1).

Cinco niños presentaron complicaciones transitorias (21%): dos linforragias postoperatorias y una paresia del elevador de la escápula tras cirugía, y dos síndromes febriles tras inyección de OK-432. No hubo complicaciones permanentes.

Tras un seguimiento medio de 5 años (rango 3-16), doce niños se curaron (52,2%) y ocho presentaban enfermedad residual estable (34,8). En tres no se logró la curación (13,0%); estos pertenecían al grupo sometido a cirugía, lo cual supone una diferencia clínicamente relevante, aunque no estadística (p=0,103). En estos tres se planteó la posibilidad de realizar escleroterapia o cirugía de resección parcial; sin embargo, la sintomatología residual no era significativa por lo que el niño y sus tutores optaron por mantener una actitud expectante con seguimiento clínico y radiológico.

Durante décadas, la cirugía fue el tratamiento de elección de las malformaciones linfáticas. No obstante, la elevada tasa de complicaciones relacionadas con este abordaje y que más del 50% de los pacientes presentaban enfermedad residual tras la intervención, ha hecho que el tratamiento farmacológico y la escleroterapia parezcan alternativas más eficaces y seguras en la actualidad^3^^,^^4^. Nuestra experiencia ha sido similar: hemos abandonado progresivamente el tratamiento quirúrgico como opción de primera línea, reservándolo solo para casos que requieren una rápida actuación y una resolución a corto plazo como, por ejemplo, el compromiso de la vía aérea. Inicialmente se reemplazó por la esclerosis con OK-432, tratamiento refrendado en la literatura por su aplicación segura y alta eficacia a largo plazo (en torno al 90%), que actualmente ha caído en desuso debido al síndrome febril que suele aparecer tras su inyección^5^, como se observó en dos de nuestros pacientes.

Con frecuencia, las malformaciones linfáticas se asocian a variantes patogénicas en diferentes vías de señalización^6^. Una de las mutaciones más documentadas involucra la molécula diana de la rapamicina (*mTOR*) y, como consecuencia, la activación de forma descontrolada de la linfangiogénesis^6^. Se ha demostrado que el uso de sirolimus, un inhibidor de *mTOR*, consigue una respuesta global en diversas anomalías vasculares del 72% en forma tópica y hasta del 85% con la administración oral, por lo que esta molécula se ha posicionado como una opción terapéutica segura y efectiva en pediatría^6^. En nuestro caso, el uso de sirolimus en anomalías vasculares es una indicación especial no autorizada en ficha técnica por lo que, con la derivación de algunos casos a un centro de referencia para su utilización, se ha conseguido demostrar también su alta seguridad y eficacia.

**Figura 2:**
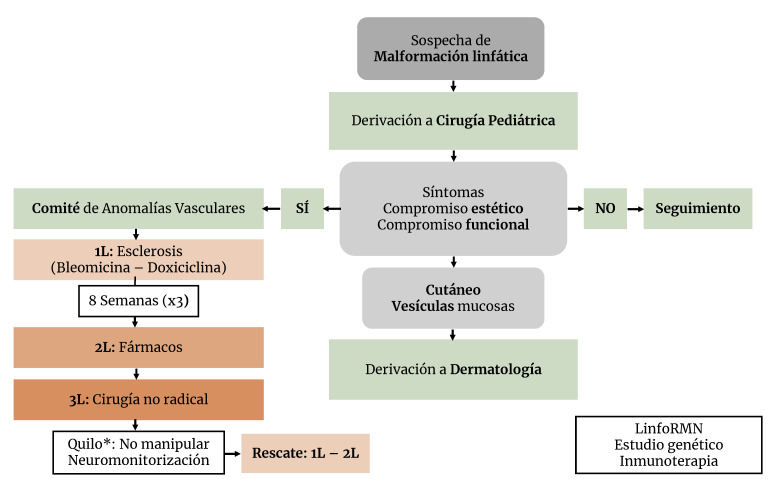
Protocolo de tratamiento ante la sospecha de malformación linfática.

Asimismo, hemos introducido la punción ecoguiada en el quirófano, lo cual ha permitido optimizar la escleroterapia. Además, la creación de un comité multidisciplinar de anomalías vasculares ha mejorado los resultados obtenidos gracias a la toma de decisiones de forma conjunta. Estas lecciones nos han llevado a desarrollar un protocolo de atención a las malformaciones linfáticas que engloba la observación, la esclerosis, los fármacos y la cirugía (Fig. 2). Se recomienda su uso de manera escalonada y en ese orden, teniendo en cuenta que se trata de terapias complementarias y que cada caso particular requiere una terapia o la combinación de estas para conseguir la máxima eficacia.

1L: primera línea (8 semanas entre cada sesión de escleroterapia); 2L: segunda línea; 3L: tercera línea; #: dosis empleadas de Bleomicina (15 UI): 0,5 (UI) por Kg de peso en niños <1 año, máximo 10 mg por sesión, y 1 mg (UI) por Kg de peso en niños ≥1 año, máximo 15 mg por sesión; *: probabilidad de trastornos de la conducción linfática como la anomalía linfática generalizada (GLA).
